# What Is the Most Dangerous Time of Birth for Uncomplicated First-Time Mothers and Their Neonates in a Tertiary Obstetric Center?

**DOI:** 10.1155/jp/6700829

**Published:** 2025-11-03

**Authors:** L. Steinkasserer, J. Hachenberg, A. Biermann, C. von Kaisenberg, P. Hillemanns, L. Brodowski

**Affiliations:** Department of Gynecology and Obstetrics, Hannover Medical School, Hannover, Germany

**Keywords:** maternal outcome, off-hour hospital care, perinatal outcome, time of birth

## Abstract

**Background:**

Whether the timing of birth affects fetal or maternal outcomes is still controversial. Compared with multiparae, primiparae are a special obstetric group that are more likely to develop labor abnormalities that require intervention. The aim of this study was to investigate the time of delivery and perinatal morbidity with a focus on uncomplicated but first-time pregnancies.

**Methods:**

This retrospective study analyzed all births of uncomplicated first-time mothers who intended vaginal delivery. The delivery times were clustered into 2-h intervals and by day of the week. A 5-min Apgar score ≤ 7, an umbilical artery pH value < 7.10, and BE > − 12 were defined as surrogate markers for perinatal morbidity. Other markers examined included the occurrence of a pathological cardiotocogram, the performance of fetal blood analysis via scalp sampling, admission to the neonatal intensive care unit (NICU), the mode of delivery, or the occurrence of labor arrest.

**Results:**

A total of 586 women who intended vaginal delivery were analyzed. The distribution of timepoints of birth divided into 2-h intervals corresponded to a normal distribution (*p* = 0.97). The probability of having an arterial umbilical pH value < 7.1 was highest on Friday between 2:00 and 4:00 PM (*p* = 0.035). A base excess below − 12 was most frequent on Sunday between 4:00 and 6:00 AM (*p* = 0.027). Fetal blood analysis via scalp sampling was performed less frequently than expected on the weekend (Saturday *p* = 0.031; Sunday *p* = 0.046), whereas the distribution of mode of delivery did not differ across the investigated periods.

**Conclusions:**

We detected timepoints when laboratory signs of increased fetal distress were more frequent; although there was no difference in peripartal monitoring, the decision to perform a scalp blood gas analysis or the mode of delivery changed during these periods. On weekends, fetal blood analysis via scalp sampling was carried out less frequently than expected. An increased rate of severe asphyxia or a worse 5-min APGAR was not observed in the neonates at any timepoint. Primiparae should not be underestimated as a supposedly uncomplicated group, as they can present a particular obstetric challenge.

## 1. Background

Birth policies worldwide aim to improve and ensure the safety of perinatal care [1, 2]. Reducing fetal morbidity, particularly perinatal asphyxia, is a core part of modern obstetrics. Hypoxic–ischemic encephalopathy due to perinatal asphyxia is one of the main causes of fetal morbidity worldwide [3]. The indications of neurological developmental delays after suffering peripartum hypoxia are fortunately rare, but the risk of permanent damage is higher after ischemic encephalopathy [4].

Blood gas analysis from the umbilical artery and assessment of the newborn using the APGAR score are suitable for adequate assessment of neonatal short-term outcomes [5, 6]. The rate of admission to the neonatal intensive care unit (NICU) correlates with these values. The assessment of Cardiotocographies (CTGs) helps to assess fetal constitution during birth and has a major influence on the management of labor and ultimately the mode of delivery.

Several studies have shown that out-of-hour births, i.e., births during evening and night shifts or on weekends, are more often associated with poorer perinatal outcomes than are births during regular working hours [7–9]. In addition to perinatal mortality, these studies examined fetal injury and early neonatal death. Some studies have questioned the quality of care, work organization, and availability of resources if there is a higher incidence of adverse perinatal outcomes in out-of-hours care [7]. However, not all studies that have examined the effect of the timing of labor on perinatal morbidity have reported a higher risk of adverse perinatal outcomes and therefore have been inconsistent.

A few years ago, we retrospectively examined all births in our clinic over a period of 14 years to determine whether there was increased perinatal morbidity at night and on weekends. A multivariate regression analysis revealed that delivery at night and on the weekend was a prognostic marker for an umbilical artery pH below 7.1 [10].

Understanding the contributing factors for perinatal morbidity is the key to reduce the number of adverse perinatal outcomes. Compared with multiparae, primiparae are an obstetric high-risk group that are more likely to develop labor abnormalities that require intervention, even in healthy, uncomplicated first-time mothers. More uterine force is required to overcome resistance in the reproductive tract, and the uterus tends to be less effective in maintaining uterine contractions. The birth phases are prolonged, and the risk of fetal compression is increased [11]. Caring for these pregnancies always requires expertise and special attention.

Therefore, the aim of this study was to investigate whether there is a connection between the time of delivery and perinatal morbidity with a focus on uncomplicated but first-time pregnancies at a tertiary obstetric center with maximum care. The time of birth will therefore be examined in more detail in relation to the time of day and day of the week.

## 2. Methods

### 2.1. Study Design

This is a retrospective data analysis of all births of uncomplicated first-time mothers who intended vaginal delivery at the Medical School of Hannover from January 1, 2020, to December 31, 2023. Retrospective data from 586 women who gave birth were used.

An uncomplicated first-time mother was defined according to the following criteria: an unremarkable pregnancy course without detected pregnancy or birth risks, an internally healthy pregnant woman with a body mass index (BMI) at the beginning of pregnancy of less than 30 kg/m^2^ without previous abdominal surgeries, a eutrophic fetus with a normal fetus Dopplers in a cephalic position without malformations, a time between rupture of the membranes and birth of a maximum of 24 h, and a gestational age at birth between 37+0 and 42+0 weeks of gestation without induction of labor or planned cesarean section. All included women presented at the onset of labor with the intention of vaginal delivery.

Depending on the course of labor, the actual mode of delivery could include spontaneous vaginal delivery, operative vaginal delivery (e.g., vacuum extraction), or secondary cesarean section. These outcomes were documented and considered in the analysis.

A total of 10,734 children were born in our clinic during the investigated period. The general cesarean section rate was 29.8%, and 5.9% gave operative vaginal birth. A total of 9834 pregnant women with cephalic position presented to the clinic to plan birth. Of these, 9248 (94.0%) patients were excluded during the recruitment process since these patients met the exclusion criteria. The final study population comprised a total of 586 cases (6.0%). Only fetuses in cephalic presentation without malpresentation or rotational abnormalities were included. Cases with breech presentation, transverse lie, or significant rotational abnormalities were excluded to ensure a homogeneous low-risk cohort. The maternal and neonatal characteristics are shown in Table 1.

A 5-min Apgar score ≤7, an umbilical artery pH value < 7.10, and BE > − 12 were defined as surrogate markers for perinatal morbidity. Other markers examined included the occurrence of a pathological cardiotocogram, the performance of fetal blood analysis via scalp sampling, admission to the NICU, the mode of delivery or the occurrence of labor arrest. Planned cesarean section was defined as a cesarean section after the intended vaginal birth approach.

Umbilical artery blood gas analysis (including pH and BE) was performed immediately after delivery using the ABL800 FLEX blood gas analyzer, which is the standard device used in our delivery unit. In case of intrapartum fetal blood analysis by scalp sampling, the procedure was as follows: Initially, the fetal scalp was carefully disinfected under direct visualization using an amnioscope or speculum. A small incision was then made until capillary blood began to flow. The blood was collected in a heparinized capillary tube and subsequently analyzed using the ABL800 FLEX blood gas analyzer.

During the study period, there was a 24-h medical on-call service in the women's clinic at Hannover Medical School. The midwives worked in three shifts. The delivery times were clustered into 2-h intervals and by day of the week.

Before the data were collected, the ethics committee was asked to evaluate the study (No. 11331_BO_K_2024). All methods were carried out in accordance with relevant guidelines and regulations.

### 2.2. Statistical Analysis

The data were assembled in a databank and analyzed using Microsoft Excel 2021 (version 16.56; Microsoft Corp., Redmond, Washington, United States). Statistical analyses were performed using GraphPad Prism 9 software (GraphPad Software, Inc.) and IBM SPSS Statistics 28 (IBM SPSS Software). The Shapiro–Wilk normality test was used to test for the normal distribution of epidemiological data. Unpaired *t* tests and Mann–Whitney tests were used as appropriate. The chi-square test of independence was used to describe the expected and observed events and to test for statistically significant differences (*p* < 0.05). In the case of statistically significant results, the effect size for a chi-square test in model comparison was calculated using Cohen's w (omega).

## 3. Results

### 3.1. Patient Characteristics and Overall Outcome

Maternal and neonatal characteristics are shown in Table 1. All women were primiparous. The average age of the women who gave birth was 29 (± 4.5). The mean BMI before pregnancy was 22.9 kg/m^2^ (± 2.7). On average, the patients gave birth on due date (39.97 ± 1.1 weeks of gestation) to a 3445 g (± 404) weighing neonate. The time between the rupture of the membranes and delivery was approximately 8 h.

A total of 204 (34.8%) women had a perineal tear. Of these, 68 (11.6%) were grade I, 116 (19.8%) were grade II, 18 (3.1%) were grade III, and 2 (0.3%) were grade IV. A total of 120 episiotomies were performed (20.5%). Birth injuries, defined as soft tissue injuries other than perineal tears, affected 239 of the women (40.8%). Of these, 12 were high-grade vaginal or cervical tears (2.1%). Perinatal vaginal hemorrhage with blood loss of more than 500 mL occurred in 44 patients (7.5%). In 15 patients (2.6%), manual placental abruption was needed. The average hospitalization time for mothers and neonates was 2.5 days.

An arterial cord blood pH less than 7.1 was observed in 33 patients (5.6%), a base excess less than − 12 was detected in 31 cases (5.3%), and a 5-min APGAR less than or equal to 7 was detected in 11 cases (1.8%). There were no cases of perinatal asphyxia with arterial cord blood pH values less than 7.0. Twelve neonates (2.1%) and none of the mothers were transferred to intensive care units (ICUs).

In all cases with pH values below 7.10, routine cranial ultrasound and neurological assessment by a pediatric neurologist were performed during the first days of life. All findings were age-appropriate and unremarkable. One neonate developed a cephalohematoma after vacuum extraction, which was monitored clinically and sonographically without signs of intracranial involvement. A pathological CTG sub partu was found in 57 cases (9.7%), and fetal blood analysis via scalp sampling was performed in 60 cases (10.2%). Failure to progress in the first stage of labor was observed 12 times (2.0%), whereas failure to progress in the second stage of labor occurred 29 times (4.9%). A planned cesarean section was performed in 62 patients (10.6%), and a vaginal operative delivery was performed in 92 patients (15.7%).

### 3.2. Distribution of Births by Time and Day of the Week

The delivery times were clustered by day of the week and in 2-h intervals.

A total of 77 children were born on Monday (13.1%), 83 on Tuesday (14.2%), 93 on Wednesday (15.9%), 82 on Thursday (14.0%), 94 on Friday (16.0%), 75 on Saturday (12.8%), and 82 on Sunday (14.0%). The distribution of births across days corresponded to a normal distribution (*p* = 0.26). A total of 73.2% of all births occurred on a weekday (*n* = 429), and 157 (26.8%) took place over the weekend.

Between 0:00 AM and 2:00 AM, 51 children were born (8.7%), 46 between 2:00 AM and 04:00 AM (7.8%), 60 between 4:00 AM and 06:00 AM (10.2%), 27 between 6:00 AM and 08:00 AM (4.6%), 44 between 8:00 AM and 10:00 AM (7.5%) and 41 between 10:00 AM and 12:00 AM (7.0%). Between 12:00 AM and 02:00 PM, 39 neonates were born (6.6%), 51 between 2:00 PM and 04:00 PM (8.7%), 55 between 4:00 PM and 06:00 PM (9.4%), 46 between 6:00 PM and 08:00 PM (7.8%), 55 between 8:00 PM and 10:00 PM (9.4%), and 71 between 10:00 PM and 00:00 AM (9.4%). The distribution of births divided into 2-h intervals corresponded to a normal distribution (*p* = 0.97).

### 3.3. Distribution of Births With an Arterial Cord Blood pH < 7.1, Base Excess ≤ − 12 or a 5-min Apgar Score ≤ 7 Depending on the Day and Time of Delivery

**Figure 1:**
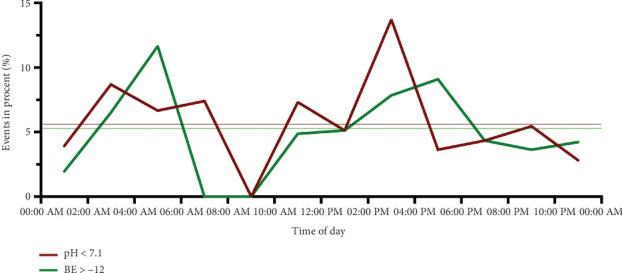
Distribution of births with an arterial cord blood pH < 7.1, base excess ≤ − 12 depending on the time of delivery.

**Figure 2:**
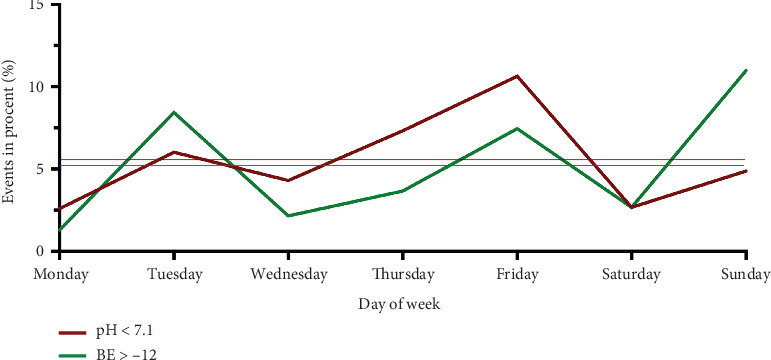
Distribution of births with an arterial cord blood pH < 7.1, base excess ≤ − 12 depending on the day of delivery.

An arterial cord blood pH value < 7.1 was found in 33 births (5.6%), and a base excess < − 12 was recorded in 31 cases (5.3%). Twelve (36%) of the births with a pH value < 7.1 occurred during the day. Accordingly, the remaining 21 (63%) births with a pH < 7.1 occurred at night (Table 2). The probability of having a pH value < 7.1 was highest between 2:00 and 4:00 PM (Figure 1). Here, the seven observed events were significantly different from the 2.9 expected events (*p* < 0.014). Similarly, the probability of a BE below − 12 was increased at this time, albeit not significantly. Between 4:00 and 6:00 AM, the probability of a BE below − 12 was highest and was observed significantly more frequently than expected. In terms of the day of the week, the probability of a pH below 7.1 was highest on Friday (*p* = 0.035), and that of a BE below − 12 was highest on Sunday (*p* = 0.027) (Figure 2). These results were statistically significant. A 5-min APGAR of 7 or less was rare, with 11 events overall. A significant difference between the expected and observed events could not be detected either at a specific time or on a specific day of the week.

### 3.4. Distribution of Occurrence of a Pathological CTG or Fetal Blood Analysis by Scalp Sampling Depending on the Day and Time of Delivery

**Figure 3:**
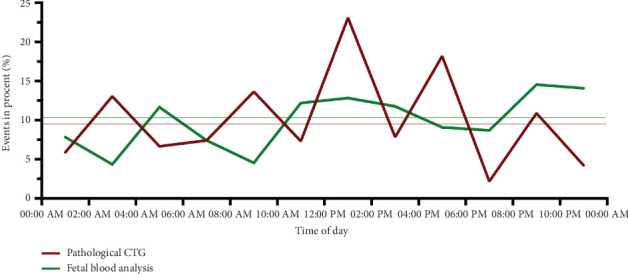
Distribution of occurrence of a pathological CTG or fetal blood analysis by scalp sampling depending on the time of delivery.

**Figure 4:**
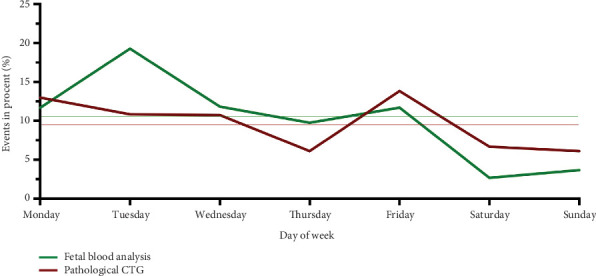
Distribution of occurrence of a pathological CTG or fetal blood analysis by scalp sampling depending on the day of delivery.

A subpartum CTG was obtained from all women. A total of 9.7% (*n* = 57) had a pathological CTG according to the FIGO classification. The CTG was most frequently abnormal between 12:00 AM and 2:00 PM (*p* = 0.005) and between 4:00 and 6:00 PM (*p* = 0.034) (Table 3). There was no difference in the frequency of pathological CTG between different days of the week (Figures 3 and 4).

In 60 (10.2%) cases, fetal blood analysis via scalp sampling was performed. These were most frequently performed on Tuesdays (*p* = 0.007) and carried out less frequently than expected on weekends (Saturday *p* = 0.031; Sunday *p* = 0.046). There was no statistically significant difference in the 2-hour intervals (Figures 3 and 4).

### 3.5. Distribution of Planned Cesarean Sections, Operative Vaginal Births, and Failure to Progress in the First or Second Stage of Labor Depending on the Day and Time of Delivery

**Figure 5:**
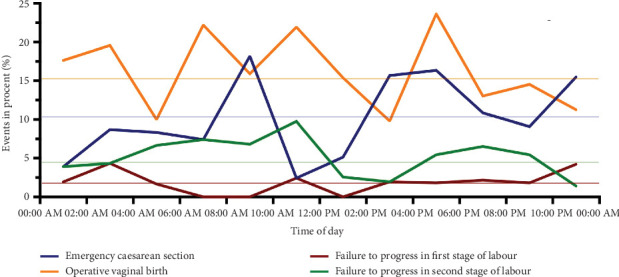
Distribution of planned cesarean sections, operative vaginal births, and failure to progress in the first or second stage of labor depending on the time of delivery.

**Figure 6:**
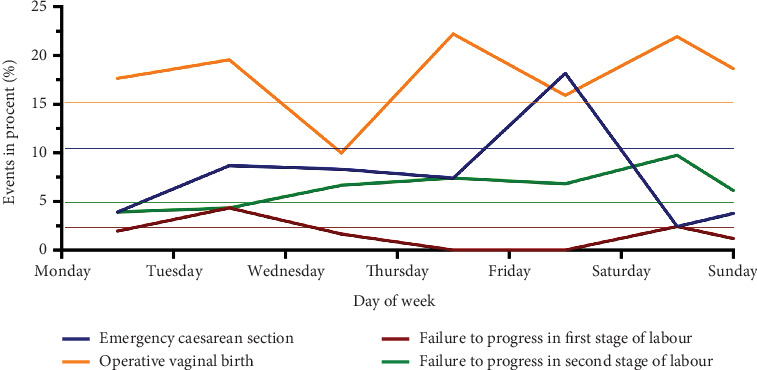
Distribution of planned cesarean sections, operative vaginal births, and failure to progress in the first or second stage of labor depending on the day of delivery.

Forty-one (7%) women failed to progress while giving birth. Twelve (29.2%) of them were in the first stage of labor, and 29 (70.8%) were in the second stage of labor (Table 4). No statistically significant differences between the number of days of the week and the time of day were found (Figures 5 and 6).

Planned cesarean sections were required for 62 (10.6%) of all the women (Table 4). There were no statistically significant differences between days of the week and time of day (Figures 5 and 6).

In 92 women (15.7%), vacuum or forceps were used to deliver the neonate (Table 4), without statistically significant differences between days of the week and time of day (Figures 5 and 6).

## 4. Discussion

Modern obstetrics strives to minimize poor neonatal and maternal outcomes. This involves the use of specific tools, such as fetal blood gas analyses or APGAR scores, to detect poor outcomes after birth. Several studies have shown that out-of-hour births, i.e., births during evening and night shifts or on weekends, are more often associated with poorer perinatal outcomes than are births during regular working hours [7–9]. However, the results have been inconsistent.

The aim of this retrospective study was to determine whether the time of birth influences neonatal and maternal outcomes in healthy normal-weight primiparas in a tertiary obstetric center. According to the umbilical artery pH value, the most dangerous time of birth was Friday between 2 and 4 PM. This is in accordance with the measured BE > − 12 and 5-min Apgar score ≤ 7, even if the APGAR values were not significantly different. These findings are in line with the results of studies that were able to show a low correlation between the umbilical artery pH/BE value and the 5-min APGAR score [12, 13].

The CTG is used to monitor fetal heart rate and, as expected, fetal well-being peripartum. On the basis of the data collected, the frequency of pathological CTGs and the decision to perform a blood gas analysis via scalp sampling in response remained the same over most investigated timepoints but was carried out less frequently than expected on weekends. However, it was not possible to prevent all poor perinatal outcomes. Moreover, poor outcomes occurred more frequently at certain times. At all times, there was an even distribution of modes of delivery. As shown in the literature, blood gas analysis via scalp sampling may avoid unnecessary interventions such as cesarean section or operative vaginal delivery but is poorly suited for confirming or excluding safe fetal hypoxia during labor [14, 15].

In conclusion, we detected timepoints, primarily Friday between 2:00 and 4:00 PM and Sunday between 4:00 and 6:00 AM, when laboratory signs of increased fetal stress were more common, despite the lack of difference in peripatal monitoring, the decision to perform scalp blood gas analysis or the mode of delivery was initiated during these periods. On weekends, fetal blood analysis via scalp sampling was carried out less frequently than expected. An increased rate of severe asphyxia or a worse 5-min APGAR was not observed in the neonates at any timepoint.

Several studies have investigated the time of birth and its correlation with neonatal outcomes. Snowden et al. reported that weekend births are a surrogate marker for perinatal complications. They emphasize that there may be differences in how well hospitals cope with high workloads [16]. A large British observational study examined the outcomes of mothers and newborns by day of the week in more than one million births. The outcomes of women and neonates hospitalized or born on weekends were significantly worse. No consistent relationship was found between outcomes and staffing levels [17]. A Greek study, which included 8572 women who gave birth in a private health facility, revealed that the lowest number of births occurred on Monday, Saturday, and Sunday. Furthermore, they demonstrated that first-time mothers with a singleton cephalic pregnancy at 37 weeks of gestation were 73% more likely to deliver by cesarean section if they gave birth between 8 AM and 4 PM than if they gave birth between 0 AM and 8 AM. The authors reported that multiparous women with a single cephalic presentation were 16.7% more likely to give birth by cesarean section in the morning than at night [1]. Gijsen et al. reported that both the induction of labor and planned cesarean deliveries at night were associated with an increased risk of adverse perinatal outcomes compared with similar daytime deliveries, whereas weekend deliveries were not associated with an increased risk compared with weekday deliveries [8].

In contrast, some studies have reported no difference in maternal or neonatal outcomes between different timepoints of birth [18–22]. Caughey et al. divided the time of delivery into day, evening, and night without finding significant differences in neonatal outcomes across 53,184 deliveries [21]. A US study investigated the effect of giving birth at night and on weekends. The study included 11,137 cases without finding a significant effect of the time of birth on the risk of mortality and morbidity [20]. Aiken et al. reported no effect on maternal or neonatal morbidity for weekend births [23].

In our study, the 2–4 PM on Friday and the 4–6 AM on Sunday were the periods when laboratory signs of increased fetal compression were more common than expected. The period of Friday 2–4 PM represents the transition from daytime medical services to weekend on-call services in our unit. A possible explanation for the higher incidence of fetal compression at this time could result in less attentive fetal monitoring and therefore misinterpretation of a pathological CTG without considering further arrangements. The probability of having a pH value < 7.1 was highest between 2:00 and 4:00 on Friday (*p* = 0.035). The greatest difference between the observed and expected BEs below − 12 was found on Sunday (*p* = 0.027). On weekends, fetal blood analysis via scalp sampling was carried out less frequently than expected. The weekend effect describes the effect of a higher mortality rate from various diseases in patients admitted on weekends than on those admitted on weekdays. Previous studies in different countries have shown a relatively high mortality rate for several diseases [17]. On the weekend, medical care is provided by fewer and often younger staff. Studies have shown that patient length of stay and mortality are influenced by specialized and experienced nursing staff. A young workforce is often associated with less expertise [24, 25]. This may explain the poorer neonatal and maternal outcomes associated with the transition from daytime to weekend. Some studies have shown that the educational value of night and weekend work is low. The working hours of junior physicians should be limited, and constant supervision on site should be ensured [26, 27].

To minimize potential confounding factors and ensure a homogenous study population, we intentionally focused on uncomplicated first-time pregnancies with clearly defined inclusion criteria. Primiparous women are known to represent a distinct obstetric subgroup, with higher rates of labor abnormalities, longer duration of labor, and increased likelihood of obstetric interventions compared to multiparous women—even in otherwise low-risk settings. Therefore, restricting the analysis to primiparae allowed for a more precise examination of temporal effects on perinatal morbidity without the influence of prior birth history.

Furthermore, we excluded women with a BMI ≥ 30 kg/m^2^, as maternal obesity is an established independent risk factor for adverse obstetric and neonatal outcomes. Including such cases would have introduced significant clinical variability that could have obscured the specific associations we aimed to investigate.

By limiting the study population to term pregnancies without preexisting medical conditions or pregnancy complications, we sought to create a clinically well-defined baseline cohort. This approach strengthens the internal validity of the study and enables a more meaningful interpretation of whether time-of-delivery itself may be associated with subtle shifts in perinatal outcomes, independent of major maternal or fetal risk factors. In a tertiary obstetric center with a maximum care setting, where women and fetuses with complex preexisting conditions are treated, this group initially appears to require less support during birth. However, this group represents a particular obstetric challenge. When labor is significantly longer, abnormalities are observed more frequently and require more interventions in primiparous women [28]. This may be a reason for poorer neonatal outcomes. In our study, 41 (7%) women failed to progress while giving birth. The rate of cesarean section appears to be higher in primiparous women than in multiparous women. A study by Williams et al. investigated 18,946 deliveries and reported a cesarean section rate of 21.3% in primiparous women compared with 14.8% overall [29]. Our rate of cesarean sections was 10.6% and therefore significantly lower, but owing to the strict exclusion criteria, our collective had a normal maternal BMI, fetal head circumference in the normal range and normal birth weight, which are factors associated with successful vaginal delivery in primiparous women [11].

Even though our study in primiparae revealed special timepoints with increased risk of laboratory signs of fetal stress, a low arterial umbilical pH or BE was a rare event, which uncommonly increased perinatal morbidity, especially since the APGAR values of the affected neonates were not significantly worse. Nevertheless, it is important for the obstetric team to know the risk of a possible poor neonatal outcome in primiparous women to minimize possible causative factors. It is possible that team training programs could improve neonatal and maternal outcomes [30]. Currently, the use of artificial intelligence (AI) and computer analysis to interpret CTG during labor does not improve neonatal outcomes. Future studies are needed to further elucidate the use of AI for monitoring sub partu [31, 32].

There are several limitations of our study. The population studied here does not represent an average of the whole population. In addition, the small sample size due to the very specific inclusion criteria should be considered. Therefore, further studies will be necessary to verify the outcome of our observation. Furthermore, the long-term outcomes of mothers and newborns were not studied. Our data were obtained by the attending obstetrician within the framework of the perinatal quality assurance initiative. In addition to the known limitations when analyzing retrospective data, coding bias could also be present in some cases.

## 5. Conclusion

We detected timepoints, primarily Friday between 2:00 and 4:00 PM and Sunday between 4:00 and 6:00 AM, when laboratory signs of increased fetal stress were more common postpartum; despite no difference in peripatal monitoring, the decision to perform scalp blood gas analysis or the mode of delivery was initiated during these periods. On weekends, fetal blood analysis via scalp sampling was carried out less frequently than expected. An increased rate of severe asphyxia or a worse 5-min APGAR was not observed in the neonates at any timepoint. Primiparae should not be underestimated as a supposedly uncomplicated group, as they can present a particular obstetric challenge.

## Figures and Tables

**Table 1:** Maternal and neonatal characteristics and outcome.

**Maternal characteristics and outcome (** **N** = 586**)**	
Age in years (mean; st.dev.)	29.1 ± 4.5
Gravida (mean; st.dev.)	1.2 ± 0.4
Para (mean; st.dev.)	0.0 ± 0.0
Miscarriage (mean; st.dev.)	0.2 ± 0.5
Induced abortion(mean; st.dev.)	0.02 ± 0.2
BMI in kg/m^2^ (mean; st.dev.)	22.9 ± 2.7
Gestational age at delivery (mean; st.dev.)	39.97 ± 1.1
Time between rupture of membranes and delivery (in days) (mean; st.dev.)	0.28 ± 0.45

**Neonatal characteristics and outcome (** **N** = 586**)**	
Fetal weight in gram (mean; st.dev.)	3445 ± 404
APGAR 1‘(mean; st.dev.)	8.76 ± 0.9
APGAR 5‘(mean; st.dev)	9.82 ± 0.6
APGAR 10‘(mean; st.dev.)	9.94 ± 0.3
PH arterial cord blood (mean; st.dev.)	7.22 ± 0.08
Perinatal asphyxia (pH arterial blood < 7.0 (*n*; %)	0 (0%)
NICU admission (*n*; %)	12 (2.1%)

**Table 2:** Appearance of pH < 7.1, base excess < − 12, and 5-min APGAR ≤ 7 for time of the day and day of the week.

**Time**	**Observed**	**Expected**	***p*** **Value**	**Cohen's w test**
pH < 7.1 (*N* = 33; 5.6% of all)				
0–2 AM	2	2.9	0.596	
2–4 AM	4	2.6	0.367	
4–6 AM	4	3.4	0.728	
6–8 AM	2	1.5	0.689	
8–10 AM	0			
10–12 AM	3	2.3	0.640	
0–2 PM	2	2.2	0.892	
2–4 PM	**7**	**2.9**	**0.014**	**0.33**
4–6 PM	2	3.1	0.521	
6–8 PM	2	2.6	0.706	
8–10 PM	3	3.1	0.955	
10–12 PM	2	4.0	0.304	
Monday	2	4.3	0.248	
Tuesday	5	4.7	0.877	
Wednesday	4	5.2	0.578	
Thursday	6	4.6	0.508	
Friday	**10**	**5.3**	**0.035**	**0.21**
Saturday	2	4.2	0.265	
Sunday	4	4.7	0.748	
Base excess < − 12 (*N* = 31; 5.3% of all)				
0–2 AM	1	2.7	0.288	
2–4 AM	3	2.4	0.709	
4–6 AM	**7**	**3.2**	**0.027**	**0.46**
6–8 AM	0			
8–10 AM	0			
10–12 AM	2	2.2	0.906	
0–2 PM	2	2.1	0.964	
2–4 PM	4	2.8	0.439	
4–6 PM	5	2.9	0.208	
6–8 PM	2	2.4	0.775	
8–10 PM	2	2.9	0.584	
10–12 PM	3	3.8	0.689	
Monday	1	4.1	0.118	
Tuesday	7	4.4	0.201	
Wednesday	2	4.9	0.176	
Thursday	3	4.3	0.509	
Friday	7	5.0	0.350	
Saturday	2	4.0	0.310	
Sunday	**9**	**4.4**	**0.024**	**0.28**
5-min APGAR ≤ 7 (*N* = 11; 1.8% of all)				
0–2 AM	0			
2–4 AM	2	0.9	0.217	
4–6 AM	0			
6–8 AM	1	0.5	0.485	
8–10 AM	0		0.157	
10–12 AM	2	0.8	0.157	
0–2 PM	1	0.7	0.752	
2–4 PM	1	1.0	0.981	
4–6 PM	2	1.0	0.337	
6–8 PM	0			
8–10 PM	1	1.0	0.974	
10–12 PM	1	1.3	0.771	
Monday	3	1.4	0.192	
Tuesday	0			
Wednesday	1	1.7	0.568	
Thursday	3	1.5	0.235	
Friday	3	1.8	0.348	
Saturday	0			
Sunday	1	1.6	0.651	

*Note:P* values < 0.05 were considered statistically significant and are shown in bold.

**Table 3:** Appearance of pathological CTG or fetal blood analysis by scalp sampling and transfer to NICU of the neonate for time of the day and day of the week.

**Time**	**Observed**	**Expected**	***p* Value**	**Cohen's w test**
Pathological CTG (*N* = 57; 9.7% of all)				
0–2 AM	3	5.0	0.354	
2–4 AM	6	4.5	0.448	
4–6 AM	4	5.8	0.423	
6–8 AM	2	2.6	0.684	
8–10 AM	6	4.3	0.382	
10–12 AM	3	4.0	0.602	
0–2 PM	**9**	**3.8**	**0.005**	**0.88**
2–4 PM	4	5.1	0.620	
4–6 PM	**10**	**5.4**	**0.034**	**0.52**
6–8 PM	1	4.5	0.084	
8–10 PM	6	5.4	0.768	
10–12 PM	3	6.9	0.118	
Monday	10	7.5	0.335	
Tuesday	9	8.1	0.732	
Wednesday	10	9.0	0.739	
Thursday	5	8.0	0.267	
Friday	13	9.1	0.180	
Saturday	5	7.3	0.371	
Sunday	5	8.1	0.255	
Fetal blood analysis by scalp sampling (*N* = 60; 10.2% of all)				
0–2 AM	4	5.2	0.572	
2–4 AM	2	4.7	0.187	
4–6 AM	7	6.1	0.715	
6–8 AM	2	2.8	0.627	
8–10 AM	2	4.5	0.213	
10–12 AM	5	4.2	0.68	
0–2 PM	5	4.0	0.595	
2–4 PM	6	5.3	0.757	
4–6 PM	5	5.6	0.779	
6–8 PM	4	4.7	0.73	
8–10 PM	8	5.6	0.292	
10–12 PM	10	7.3	0.285	
Monday	9	7.9	0.675	
Tuesday	**16**	**8.5**	**0.007**	**0.28**
Wednesday	11	9.5	0.613	
Thursday	8	8.4	0.885	
Friday	11	9.6	0.640	
Saturday	**2**	**7.7**	**0.031**	**0.33**
Sunday	**3**	**8.5**	**0.046**	**0.29**
Transfer to NICU of the neonate (*N* = 12; 2.0% of all)				
0–2 AM	1	1	0.965	
2–4 AM	2	0.9	0.271	
4–6 AM	0			
6–8 AM	0			
8–10 AM	0			
10–12 AM	2	0.8	0.201	
0–2 PM	0			
2–4 PM	2	1.1	0.360	
4–6 PM	0			
6–8 PM	1	0.9	0.952	
8–10 PM	2	1.1	0.406	
10–12 PM	2	1.5	0.647	
Monday	0			
Tuesday	2	1.7	0.816	
Wednesday	2	1.9	0.944	
Thursday	4	1.7	0.070	
Friday	1	1.9	0.501	
Saturday	0			
Sunday	3	1.7	0.314	

*Note:P* values < 0.05 were considered statistically significant and are shown in bold.

**Table 4:** Appearance of arrest in the first and second stages of labor, unplanned cesarean section, and vacuum or forceps delivery for time of the day and day of the week.

**Time**	**Observed**	**Expected**	***p* Value**	**Cohen's w test**
Arrest in the first stage of labor (*N* = 12; 2.0% of all)				
0–2 AM	1	1	0.964	
2–4 AM	2	0.9	0.271	
4–6 AM	1	1.2	0.834	
6–8 AM	0			
8–10 AM	0			
10–12 AM	1	0.8	0.860	
0–2 PM	0			
2–4 PM	1	1.1	0.949	
4–6 PM	1	1.1	0.903	
6–8 PM	1	0.9	0.953	
8–10 PM	1	1.1	0.903	
10–12 PM	3	1.5	0.196	
Monday	4	1.6	0.051	
Tuesday	0			
Wednesday	2	1.9	0.945	
Thursday	1	1.7	0.596	
Friday	3	1.9	0.435	
Saturday	0			
Sunday	2	1.7	0.817	
Arrest in the second stage of labor (*N* = 29; 4.9% of all)				
0–2 AM	2	2.6	0.724	
2–4 AM	2	2.3	0.839	
4–6 AM	4	3.0	0.554	
6–8 AM	2	1.4	0.566	
8–10 AM	3	2.2	0.580	
10–12 AM	4	2.1	0.162	
0–2 PM	1	2.0	0.485	
2–4 PM	1	2.6	0.309	
4–6 PM	3	2.8	0.877	
6–8 PM	3	2.3	0.636	
8–10 PM	3	2.8	0.877	
10–12 PM	1	3.6	0.165	
Monday	2	3.8	0.341	
Tuesday	3	4.1	0.575	
Wednesday	5	4.6	0.850	
Thursday	6	4.1	0.323	
Friday	5	4.7	0.869	
Saturday	6	3.7	0.223	
Sunday	2	4.1	0.286	
Unplanned cesarean section (*N* = 62; 10.6% of all)				
0–2 AM	2	5.4	0.121	
2–4 AM	4	4.9	0.675	
4–6 AM	5	6.4	0.568	
6–8 AM	2	2.9	0.590	
8–10 AM	8	4.7	0.102	
10–12 AM	1	4.3	0.090	
0–2 PM	2	4.1	0.267	
2–4 PM	8	5.5	0.262	
4–6 PM	9	5.8	0.165	
6–8 PM	5	4.9	0.953	
8–10 PM	5	5.8	0.716	
10–12 PM	11	7.5	0.180	
Monday	9	8.2	0.756	
Tuesday	4	8.8	0.087	
Wednesday	12	9.9	0.471	
Thursday	5	8.7	0.185	
Friday	11	10.0	0.729	
Saturday	11	8.0	0.253	
Sunday	10	8.8	0.668	
Vacuum of forceps delivery (*N* = 92; 15.7% of all)				
0–2 AM	9	8.0	0.702	
2–4 AM	9	7.2	0.471	
4–6 AM	6	9.4	0.225	
6–8 AM	6	4.2	0.352	
8–10 AM	7	6.9	0.97	
10–12 AM	9	6.4	0.271	
0–2 PM	6	6.1	0.957	
2–4 PM	5	8.2	0.228	
4–6 PM	13	8.6	0.106	
6–8 PM	6	7.2	0.620	
8–10 PM	8	8.6	0.814	
10–12 PM	8	11.1	0.305	
Monday	16	12.1	0.221	
Tuesday	12	13.0	0.756	
Wednesday	17	14.6	0.494	
Thursday	14	12.9	0.733	
Friday	14	14.8	0.830	
Saturday	11	11.8	0.806	
Sunday	8	13.0	0.129	

## Data Availability

Raw data were generated at the Department of Obstetrics and Gynecology of Hannover Medical School (MHH). The datasets used and analyzed during the current study are available from the corresponding author upon reasonable request.

## Nomenclature

NICU: Neonatal intensive care unit
CTG: Cardiotocography
BMI: Body mass index
